# The terpenes alpha-bisabolol and camphene modulate pruritus via an action on Cav3.2 T-type calcium channels

**DOI:** 10.1186/s13041-025-01196-9

**Published:** 2025-03-18

**Authors:** Flavia T. T. Antunes, Vinicius M. Gadotti, Gerald W. Zamponi

**Affiliations:** 1 Department of Clinical Neurosciences, Cumming School of Medicine, Hotchkiss Brain Institute, Alberta Children’s Hospital Research Institute, University of Calgary, Calgary, T2N 4N1 Canada; 2 School of Health Sciences, Postgraduate Program in Pharmaceutical Sciences, Universidade do Vale do Itajaí (UNIVALI), Itajaí, Brazil

**Keywords:** Alpha-bisabolol, Calcium channel, Camphene, Cav3.2, Itch

## Abstract

Alpha-bisabolol and camphene have demonstrated analgesic effects in inflammatory pain models by blocking Cav3.2 calcium channels. As the pain pathway overlaps with mechanisms for itch, and because Cav3.2 channels have been associated with itch in our previous work, we aimed to investigate the potential anti-itch effects of these two terpenes. Although both terpenes failed to show anti-pruritogenic properties when dissolved in aqueous PBS, when diluted in Hydroxypropyl-beta-cyclodextrin their bioactivity significantly increased. Both compounds significantly reduced scratching in the histaminergic itch model, whether administered subcutaneously or intraperitoneally. Camphene reduced itching in the non-histaminergic model regardless of the route of administration, whereas alpha-bisabolol did not alleviate chloroquine-induced itching. When tested in Cav3.2-/- mice, neither camphene nor alpha-bisabolol significantly reduced histamine-induced scratching behavior. This suggests that the anti-pruritic actions of these terpenes may involve Cav3.2 block to mitigate itch.

## Introduction

Itch, which affects approximately 15% of the population, can cause considerable distress, with scratching often offering only temporary relief [1]. Chronic or acute itch, whether due to dermatological or systemic conditions, is often difficult to manage and can lead to significant discomfort. Emerging data show that itch and pain share overlapping neuronal pathways, with intracellular calcium mobilization playing a central role in both conditions. T-type calcium channels, particularly Cav3.2, have been identified as crucial regulators of neuronal excitability, and they have emerged as a promising target for alleviating both itch and pain [2–4]. Blocking T-type channels has been shown to reduce scratching behavior in male and female mice, and studies in Cav3.2-/- mice suggest these animals are resistant to pruritogen-induced scratching in experimental models, further supporting the idea that Cav3.2 plays a key role in itch [3].

Terpenes are naturally occurring chemicals present in plants, and like cannabinoids, they can be found in cannabis, but they are chemically distinct [5]. Our past research has revealed that terpenes such as camphene and alpha-bisabolol did not induce analgesia in Cav3.2-/- mice in a CFA-induced pain model, suggesting that these terpenes mediate analgesia through Cav3.2 channels [6]. Given these findings, the current study aims to investigate whether these terpenes can also influence itch sensation through Cav3.2 channels, expanding our understanding of how these compounds may modulate both pain and itch pathways. We find that alpha-bisabolol and camphene are both capable of reducing pruritogen-induced scratching behavior in mice, in a Cav3.2-dependent manner. Our data open potential new avenues for targeted treatments in patients suffering from chronic itch and related disorders.

## Methods

### Animals

Eight-week-old male C57BL mice were purchased from Jackson Laboratories and allowed at least 7 days for sufficient acclimatization time before experiments. Cav3.2^−/−^ mice from breeding pairs originally obtained from Jackson Laboratories were bred in-house. They were kept in groups of a maximum of five per cage, provided with food and water ad libitum and maintained on a 12-hour light-dark cycle. The housing environment was consistently controlled at a temperature of 24–25 °C.

All experiments were approved by the institutional Animal Care Committee of University of Calgary. They were performed in accordance with animal care regulations and policies of the Canadian Council on Animal Care. They were carried out at the Animal Resource Centre at Foothills Medical Centre between 9 AM and 4 PM using different randomized cohorts of mice for each test.

### Induction and evaluation of scratching behavior

Mice were habituated for at least 20 min in an individual small plastic enclosure (12 cm x 12 cm x 15 cm). Histaminergic itch was induced by injecting Histamine dihydrochloride (Sigma-Aldrich, H7250) 100 µg/20ul, and non-histaminergic itch was modeled by injecting Chloroquine phosphate (Sigma-Aldrich, PHR1258) 200 µg/20ul. Both pruritogenic compounds were dissolved in Phosphate Buffered Saline (PBS).

Following subcutaneous injection of the pruritogenic compound at the nape, mice were immediately observed for scratching behavior. Scratching episodes were manually recorded for 30 min using a stopwatch. A scratching event was defined as the animal lifting its hind paw to touch the injection site before returning it to the floor or near its mouth [3].

### Treatments

The terpenes were dissolved in 100% dimethylsulfoxide (DMSO) and stored at -20 °C. Fresh solutions at different doses were prepared on the same day of experiments where both camphene (Sigma-Aldrich, 456055) or alpha-bisabolol (Sigma-Aldrich, W466620) were dissolved in PBS or Hydroxypropyl-beta-cyclodextrin 2.8% (HPBC) (Sigma-Aldrich, 332607). Mice were subcutaneously injected with a combination of a pruritogen and either Camphene or Alpha-Bisabolol. They were also tested for these compounds intraperitoneally 30 min before receiving histamine or chloroquine. Control conditions refer to animals treated with vehicles only.

### Statistics

Data were analyzed with Graphpad Prism 10.0. They are presented as the mean ± SEM and were analyzed using One-way analysis of variance (ANOVA) with Tukey’s post hoc or Unpaired t-test. Statistical significance was determined at the level of *p* < 0.05.

## Results and discussion

**Fig. 1:**
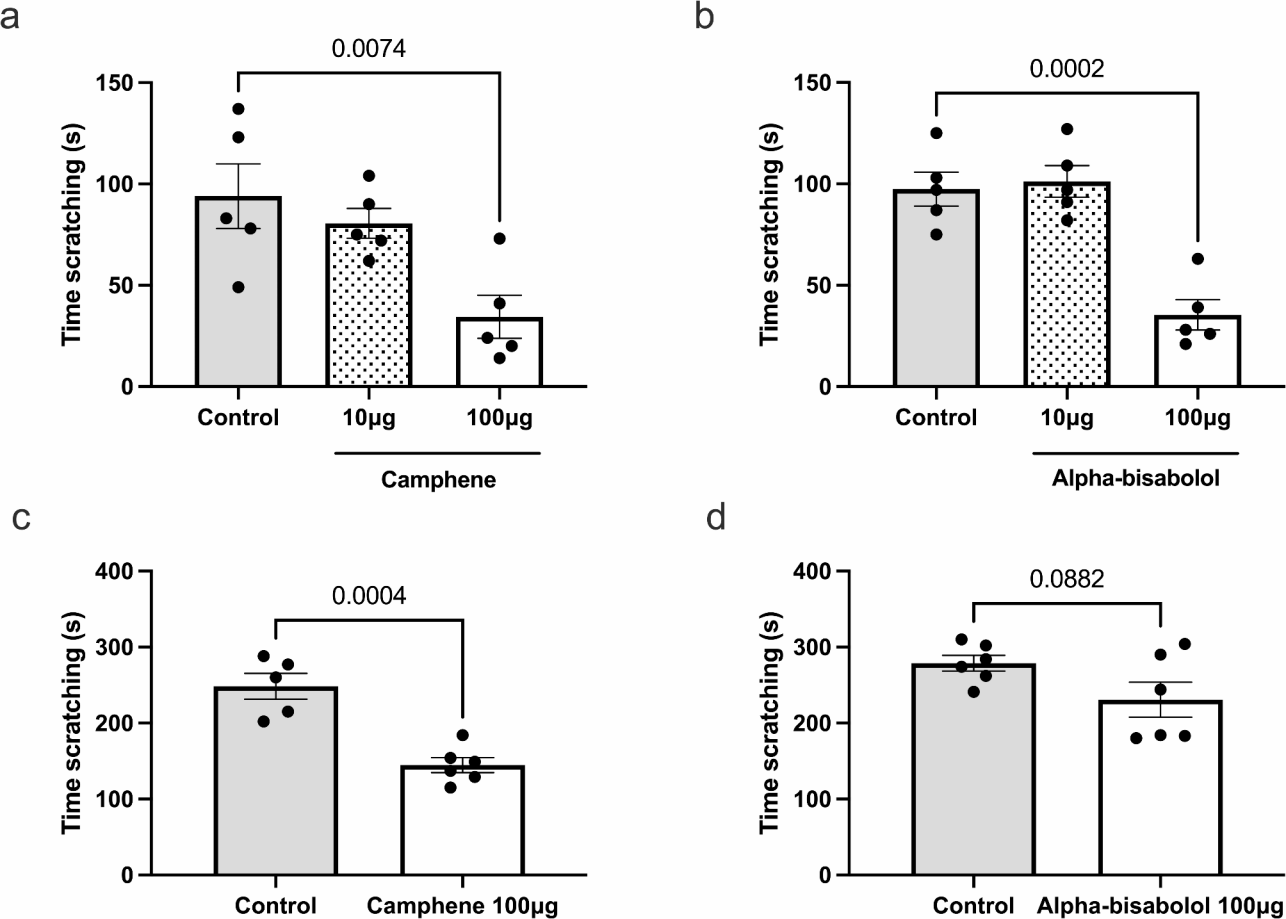
Anti-pruritic effects of terpenes injected subcutaneously in mouse models of histaminergic and non-histaminergic itch. Time scratching after different doses of (**a**) camphene or (**b**) alpha-bisabolol in histamine model. Time scratching with and without camphene (**c**) or alpha-bisabolol (**d**) at 100 µg (s.c.) in the chloroquine model. Each bar represents the mean ± S.E.M (*n* = 5–6). **p < 0.01, ***p < 0.001, ns = no significance. Panels a and b were analyzed by One-way ANOVA followed by a Tukey’s test. Panels c and d were analyzed by unpaired t-test

We first investigated the effects of different doses of camphene and alpha-bisabolol co-delivered subcutaneously (s.c.) with histamine into male wild type mice. Delivery of histamine alone induced scratching behavior in mice that lasted approximately 100 s over a 30 min observation period (Fig. 1). When we used PBS as the vehicle for the two terpenes, neither one of them was able to decrease the scratching responses evoked by histamine at concentrations varying from 1 to 100 µg (s.c.) (data not shown). When we substituted the PBS vehicle with HPBC, we observed that both camphene and alpha-bisabolol significantly reduced the time spent scratching (*p* = 0.0074 and *p* = 0.0002 respectively) induced by histamine at the highest dose tested in wild-type animals (Fig. 1a, b). We also compared PBS vs. HPBC vehicle responses in the histaminergic itch model, and found no differences in the time spent scratching (*n* = 10–13; *p* = 0.8660, t-test). For chloroquine-induced itch, camphene at 100 µg (s.c.) significantly reduced scratching time (*p* = 0.0004) (Fig. 1c), while alpha-bisabolol at 100 µg (s.c.) showed a trend towards reducing scratching time (*p* = 0.0882), but without reaching statistical significance (Fig. 1d). Based on these results, subsequent experiments were conducted using HPBC 2.8% as the vehicle.

**Fig. 2:**
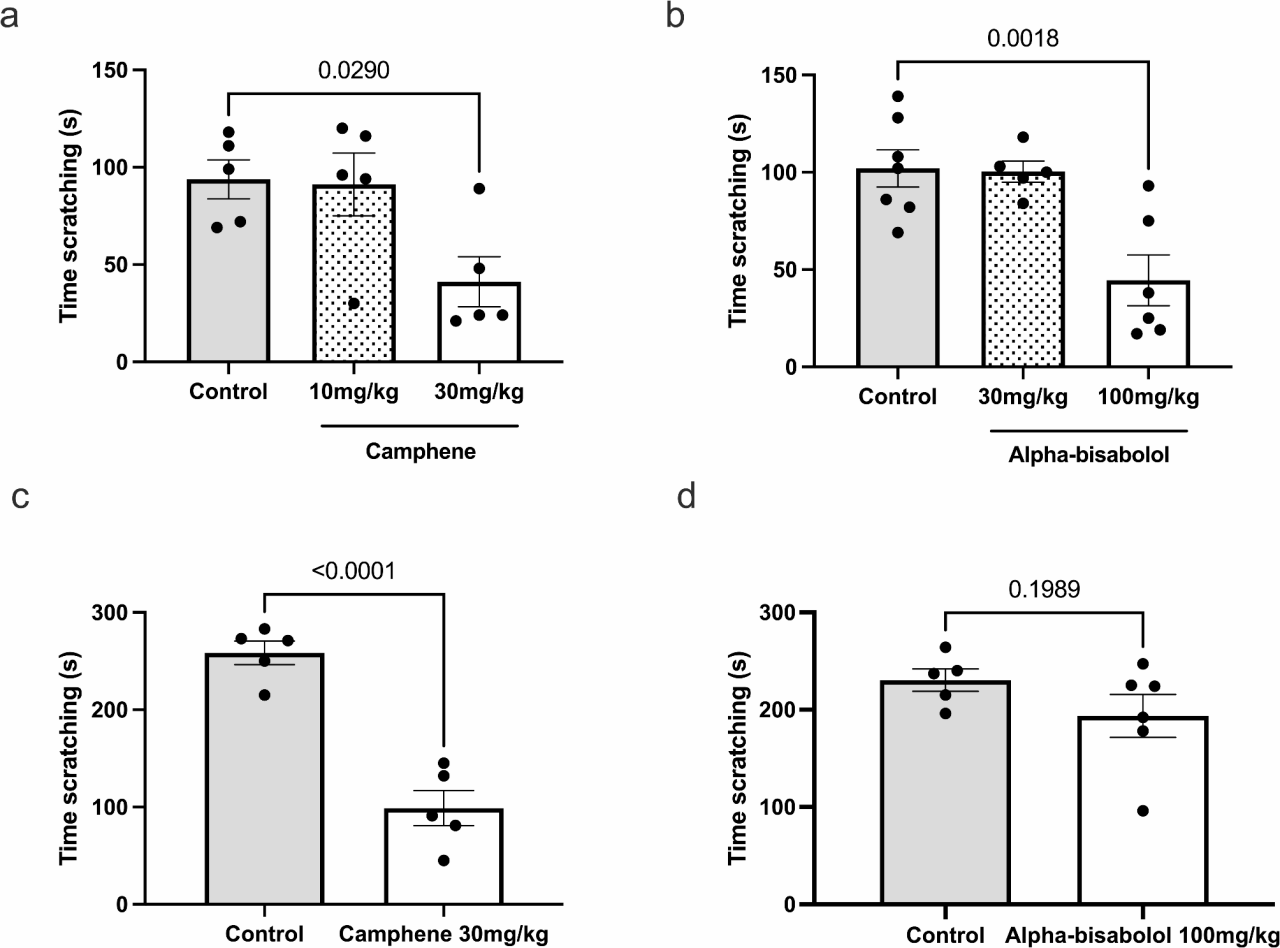
Anti-pruritic effects of terpenes injected intraperitoneally on histaminergic and non-histaminergic itch. Time scratching after different doses of (**a**) camphene or (**b**) alpha-bisabolol in the histamine model. Time scratching with and without camphene (**c**) or alpha-bisabolol (**d**) at 100 µg (i.p.) in the chloroquine model. Each bar represents the mean ± S.E.M (*n* = 5–6). *p < 0.05, **p < 0.01, ****p < 0.001, ns = no significance. Panels a and b were analyzed by One-way ANOVA followed by a Tukey’s test. Panels c and d were analyzed by unpaired t-test

Next, we tested whether the intraperitoneal delivery of terpenes could reduce the duration of itch responses. As shown in Fig. 2a, camphene at 30 mg/kg significantly reduced histaminergic scratching behavior (*p* = 0.0290). Similarly, Fig. 2b shows that alpha-bisabolol at 100 mg/kg was also effective in significantly decreasing scratching behavior (*p* = 0.0018). For non-histaminergic itch induced by chloroquine, only camphene at 30 mg/kg (i.p.) significantly reduced scratching time (*p* = 0.0001) (Fig. 2c). In contrast, alpha-bisabolol at 100 mg/kg (i.p.) did not show any significant effect on scratching time (*p* = 0.1989) (Fig. 2d).

**Fig. 3:**
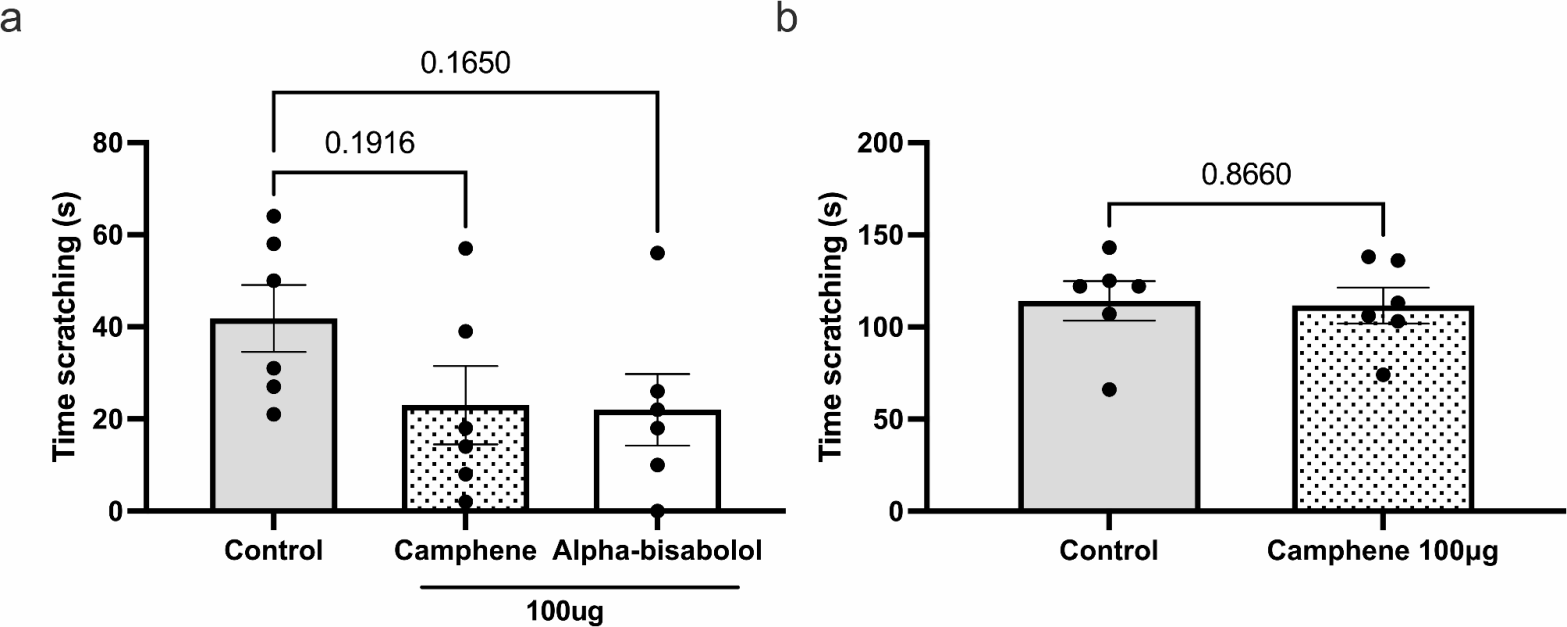
Lack of effect of subcutaneously injected terpenes against histamine or chloroquine induced itch in Cav3.2 -/- mice. (**a**) Effect of camphene and alpha-bisabolol at 100 µg (s.c.) co-delivered with histamine. (**b**) Effect of camphene at 100 µg (s.c.) co-delivered with chloroquine. Each bar represents the mean ± S.E.M (*n* = 6). Ns = no significance. Panel a was analyzed by One-way ANOVA. Panels b was analyzed by unpaired t-test

To investigate whether terpenes primarily target Cav3.2 channels to reduce scratching behavior, we administered camphene and alpha-bisabolol subcutaneously in Cav3.2-/-mice using the histamine model. As shown in Fig. 3a, Cav3.2-/- mice exhibited 60% shorter scratching times than the wild type animals (see Figs. 1 and 2), and neither camphene (*p* = 0.1916) nor alpha-bisabolol (*p* = 0.1650) produced significant additional shortening of these times in Cav3.2-/- mice. Given that alpha-bisabolol was ineffective in the chloroquine model, we tested only camphene in this model. Cav3.2-/- mice showed greatly reduced scratching behavior upon chloroquine treatment compared to wild type mice. We found no additional anti-pruritic effects upon treatment with camphene (*p* = 0.8660) in Cav3.2-/- mice (Fig. 3b). These findings suggest that these terpenes mediate their anti-itch effects via an action on Cav3.2 channels.

Cannabinoids, have previously been investigated for their potential to alleviate pruritus [7]. Terpenes, which are aromatic compounds found in cannabis (and other plants), can influence the effects of cannabinoids, often working synergistically in what is referred to as the “entourage effect” [5]. The terpenes alpha-bisabolol and camphene have already shown analgesic effects in inflammatory models of pain [6, 8–11]. The present work, to our knowledge, is the first to report anti-scratching behavior mitigated by these terpenes. Notably, both compounds failed to exhibit anti-pruritogenic properties when prepared in an aqueous PBS solution. However, when diluted in HPBC, their bioavailability significantly improved. This can be attributed to the enhanced activity of HPBC to encapsulate within the hydrophobic cavity and maintain the high lipophilicity of terpenes [12]. This finding aligns with previous studies highlighting the potential of alpha-bisabol in enhanced formulations to achieve greater analgesic effects, such as through nanoparticle delivery systems [13] or β-cyclodextrin complexes [14, 15].

Both compounds significantly reduced scratching time in the histaminergic itch model when administered either subcutaneously or intraperitoneally. Camphene was effective in reducing scratching behavior in the non-histaminergic model regardless of the route of administration, whereas alpha-bisabolol did not alleviate chloroquine-induced itch. Although the exact mechanism by which chloroquine induces itch remains unclear, it has been proposed that chloroquine acts through C fibers, modulating the itch sensation centrally. In contrast, scratching is mediated by A fibers, which temporarily suppress peripheral itch sensation [16]. Given that camphene is a monoterpene and alpha-bisabolol is a sesquiterpene [17], the differences in their chemical structure and molecular size may contribute to the observed differential effects on alleviating chloroquine-induced scratching behavior. While both terpenes exhibit a range of biological activities, and studies directly comparing their bioactivities are limited, it can be hypothesized that small terpenes like camphene, but not alpha-bisabolol, may modulate this pathway to alleviate itch.

Cav3.2-/- mice exhibited reduced scratching responses compared to wild-type controls, as reported in our previous study [2], thus again implicating these channels in itch. Our findings indicate that neither camphene nor alpha-bisabolol were able to significantly further reduce scratching responses in these Cav3.2-deficient mice. We thus conclude that camphene and alpha-bisabolol mitigate histamine-induced pruritus primarily by a blocking action on Cav3.2 channels. This fits with our previous observations that synthetic blockers of Cav3.2 channels reduce itch via these channels in both male and female mice [2]. The experiments in wild type (Fig. 1) and Cav3.2-/- (Fig. 3) mice were conducted with sub-cutaneous injection of terpenes. Assuming a local site of action, our data indicate that Cav3.2 channels contribute to the itch perhaps by initiating or facilitating action potential firing in nerve endings of sensory neurons. It is possible that the systemic delivery of the terpenes studied here may also lead to additional actions on Cav3.2 channels expressed in the dorsal root ganglia and/or the spinal cord. Because we did not test systemic delivery in Cav3.2-/- mice, we cannot rule out the possibility that the two terpenes may have additional actions on other ion channels or receptors in the itch pathway such as, for example, adenosine receptors that have been associated with antinociceptive effects of certain terpenes [18]. Nonetheless, our data further implicate Cav3.2 channels as potential molecular targets for inhibiting itch, and may suggest that topical local application of Cav3.2 inhibitors could be considered as a potential therapeutic avenue. Finally, it is also interesting to note that the consumption of cannabis has been shown efficacy in the treatment of chronic pruritus [19]. Whether these clinical effects are due to the action of terpenes, cannabinoids or both remains to be explored.

## Data Availability

The authors confirm that the data supporting the findings of this study are available within the article.
